# Mapping the dynamics of insulin-responsive pathways in the blood–brain barrier endothelium using time-series transcriptomics data

**DOI:** 10.1038/s41540-022-00235-8

**Published:** 2022-08-16

**Authors:** Zengtao Wang, Xiaojia Tang, Suresh K. Swaminathan, Karunya K. Kandimalla, Krishna R. Kalari

**Affiliations:** 1 Department of Pharmaceutics and Brain Barriers Research Center, College of Pharmacy, University of Minnesota, Minneapolis, MN USA; 2 Department of Quantitative Health Sciences, Mayo Clinic, 200 First Street SW, Rochester, MN USA

**Keywords:** Time series, Neuroscience, Software

## Abstract

Critical functions of the blood–brain barrier (BBB), including cerebral blood flow, energy metabolism, and immunomodulation, are regulated by insulin signaling pathways. Therefore, endothelial insulin resistance could lead to BBB dysfunction, which is associated with neurodegenerative diseases such as Alzheimer’s disease (AD). The current study aims to map the dynamics of insulin-responsive pathways in polarized human cerebral microvascular endothelial cell (hCMEC/D3) monolayers. RNA-Sequencing was performed on hCMEC/D3 monolayers with and without insulin treatment at various time points. The Short Time-series Expression Miner (STEM) method was used to identify gene clusters with distinct and representative expression patterns. Functional annotation and pathway analysis of genes from selected clusters were conducted using Webgestalt and Ingenuity Pathway Analysis (IPA) software. Quantitative expression differences of 16,570 genes between insulin-treated and control monolayers were determined at five-time points. The STEM software identified 12 significant clusters with 6880 genes that displayed distinct temporal patterns upon insulin exposure, and the clusters were further divided into three groups. Gene ontology (GO) enrichment analysis demonstrated that biological processes protecting BBB functions such as regulation of vascular development and actin cytoskeleton reorganization were upregulated after insulin treatment (Group 1 and 2). In contrast, GO pathways related to inflammation, such as response to interferon-gamma, were downregulated (Group 3). The IPA analyses further identified insulin-responsive cellular and molecular pathways that are associated with AD pathology. These findings unravel the dynamics of insulin action on the BBB endothelium and inform about downstream signaling cascades that are potentially disrupted due to brain insulin resistance prevalent in AD.

## Introduction

Cerebrovascular endothelium, commonly referred to as the blood–brain barrier (BBB), is instrumental in maintaining vascular response to regulate cerebral blood flow, delivering essential nutrients for sustaining brain functions, and removing toxic metabolites from the brain^1^. In addition, the BBB serves as a formidable barrier protecting the brain from circulating xenobiotics and immune challenges emanating from the periphery^2^. The BBB accomplishes these diverse functions by not functioning independently but as a crucial part of the neurovascular unit (NVU), which is organized by the precise spatial arrangement and well-coordinated communication among various cells in the cerebral vasculature (endothelial cells, pericytes, and smooth muscle cells) and the brain parenchyma (astrocytes and neurons)^3^. The molecular mechanisms regulating NVU composition and function in health and disease are only partially understood because of the paucity of molecular-level information on the less abundant, yet functionally critical cerebrovascular endothelial cells.

**Fig. 1:**
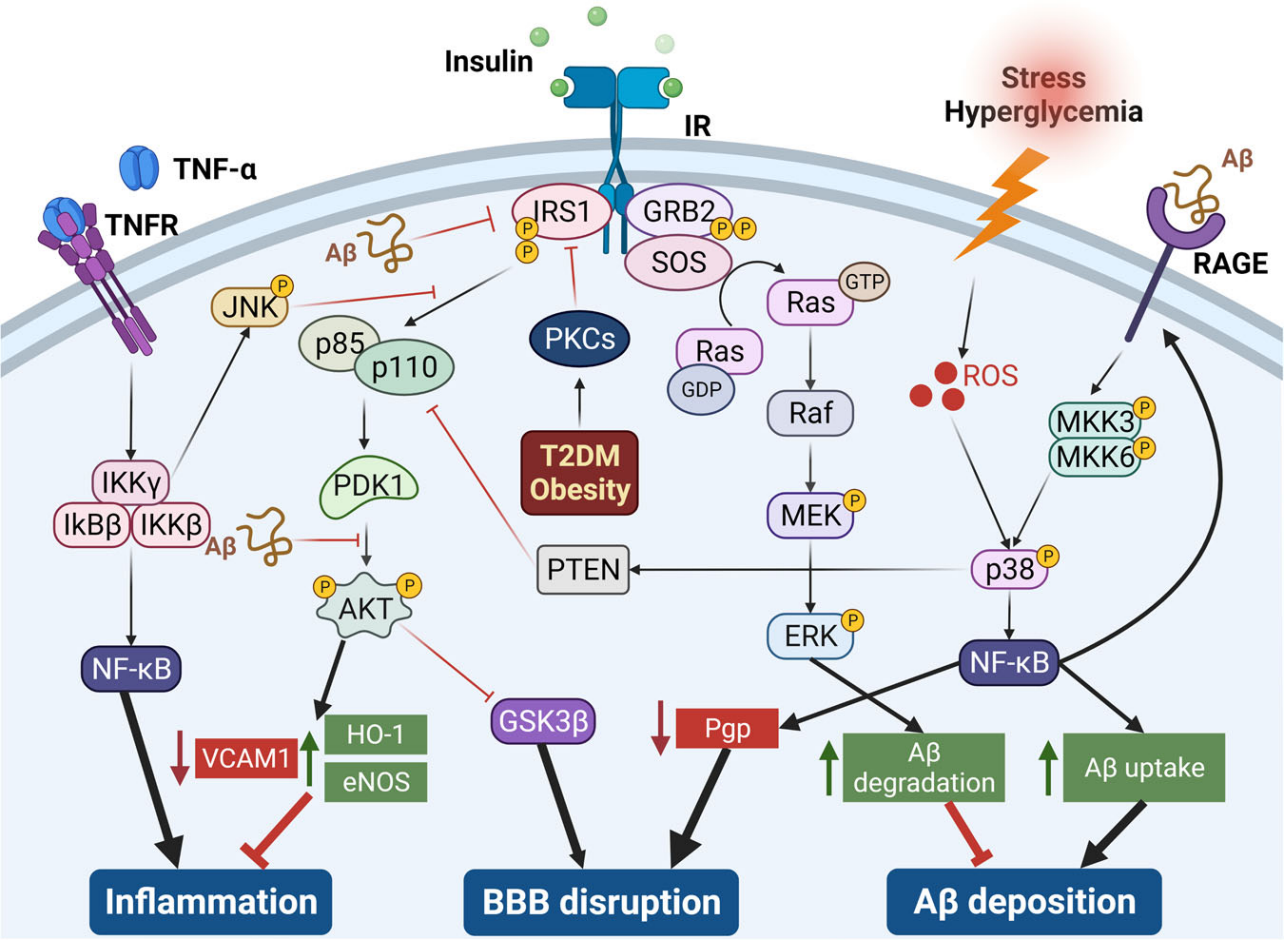
Insulin signaling pathway was found to be associated with cerebrovascular pathologies in AD including inflammation, BBB disruption, and Aβ accumulation. Inflammation: PI3K/Akt pathway mediates eNOS activation^10^ which could inhibit VCAM-1 expression^11^ in endothelial cells. BBB disruption: Inhibition of GSK3β was shown to promote tight junction stability in BBB^8^ and insulin enhanced the BBB integrity via PI3K/Akt/GSK3β pathway^48^. Aβ accumulation: Insulin exposure promoted Aβ degradation via an increase in insulin-degrading enzyme (IDE) expression in astrocytes, via activation of the ERK-mediated pathway^9^. In neurons, Aβ exposure was shown to induce insulin resistance by inhibiting IRS1^66^, PDK-dependent Akt activation^67^, and activating p38 pathway^68^. Preliminary data from our lab suggests that Aβ could have a similar effect on these pathways in BBB endothelial cells.

Studies have shown that BBB dysfunction is associated with neurodegenerative diseases such as Alzheimer’s disease (AD) and Parkinson’s disease^4,5^. Recent research has revealed the role of hyperinsulinemia and peripheral insulin resistance in AD pathogenesis^6,7^. Further, several cerebrovascular pathologies implicated in AD including BBB disruption^8^, amyloid-beta (Aβ) deposition^9^, and inflammation^10,11^ were shown to be regulated by insulin signaling, as summarized in Fig. 1. Therefore, it is crucial to characterize insulin-responsive pathways in the BBB endothelium and decipher their roles in AD. The RNA-Sequencing technology presents a powerful tool to investigate transcriptomic changes across the genome and enables us to decipher insulin’s role in the gene regulation of various cell types that orchestrate insulin action.

Given the critical regulatory role played by insulin on BBB endothelial cells, we performed RNA-Sequencing (RNA-Seq) of polarized human cerebral microvascular endothelial cell (hCMEC/D3) monolayers with and without insulin exposure. Gene expression data from the RNA-Seq experiment was analyzed with bioinformatics methods to identify molecular and cellular pathways that are responsive to insulin. In the current study, we conducted experiments at five-time points to decipher the temporal pattern of insulin response and identify early and late responding pathways activated or inhibited following insulin treatment in the hCMEC/D3 cell monolayers. Although time-series microarray analysis describing insulin action on peripheral endothelial cells has been previously reported^12^, the current study systematically investigated the transcriptional response to insulin in BBB endothelial cells using time-series RNA-Seq data.

## Results

Due to the cost of RNA-sequencing and lack of tissue samples, most gene expression studies are single snap-shot studies to identify differentially expressed genes/pathways among various groups. Biological processes are often dynamic and require temporal monitoring to decipher their modulation in health and disease. Insulin signaling pathways that muster rapid response upon stimulation and quick return to the baseline levels are prime examples of pathways requiring temporal monitoring to enable dynamic regulation of physiological responses. However, it is challenging to obtain time serial samples of BBB endothelium in vivo. Therefore, we have conducted in vitro studies using the hCMEC/D3 monolayers to capture the temporal dynamics of insulin-responsive pathways within the BBB. The RNA libraries were prepared and sequenced as described in the methods section. Raw gene expression counts were obtained, and the total read depth across samples was identified to vary between 92 and 149 million reads. Of the 19,971 genes represented in the expression data, there were 3401 genes with less than 32 counts across all ten samples. After filtering out the genes with very low expression in BBB endothelial cells, we had 16,570 genes that are available for the time-series data analysis (Supplementary Fig. 1). We determined the quantitative gene expression differences between the treatment and control at all five-time points (*t* = 10, 20, 40, 80, and 300 minutes). Differences in the gene expression between control and insulin-treated hCMEC/D3 pairs were analyzed using Short Time-series Expression Miner (STEM) method.

### Cell viability and insulin receptor expression in hCMEC/D3 monolayers

**Fig. 2:**
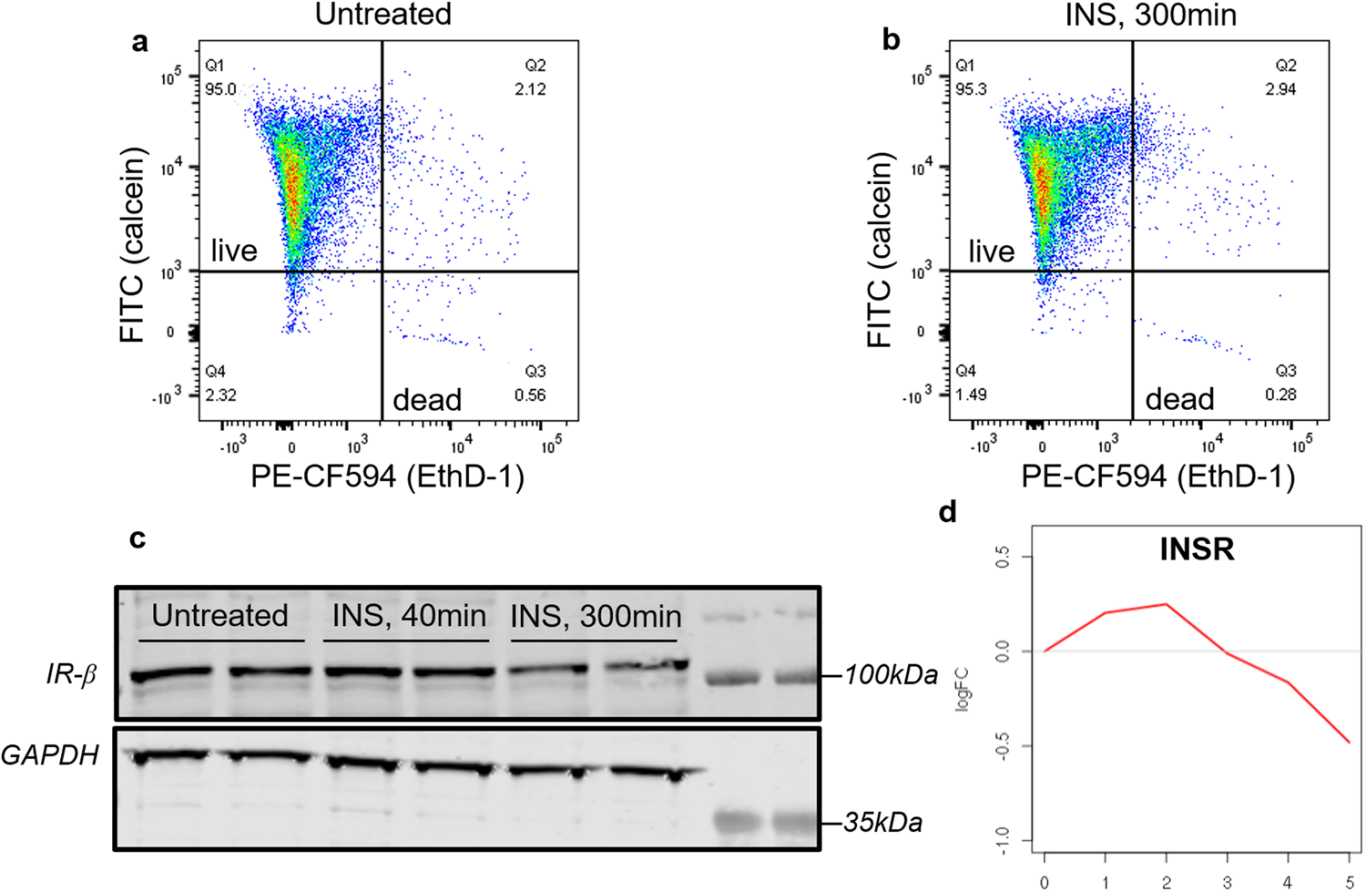
Cell viability and insulin receptor expression under current experimental condition. **a**, **b** Scatter plot of cells’ calcein/EthD-1 fluorescence showing the percentage of total counted cells within the live and dead gates in **a** untreated control and **b** insulin-treated (300 minutes) cells. **c** Immunoblots showing IR-β expression in hCMEC/D3 cells treated with insulin for 40 and 300 minutes. **d** Dynamic changes of insulin receptor gene (INSR) expression in hCMEC/D3 cells treated with insulin up to 300 minutes.

To confirm the viability of hCMEC/D3 monolayers upon insulin exposure a separate study was conducted. At the end of the last time point (300 minutes), the cells were stained by live/dead cell stains and then assayed by flow cytometry. As shown in Fig. 2a, b, there was no change in the percentage of live cell population in the untreated control versus insulin-exposed cells, thus indicating that the cells remained viable under the current experimental conditions. Further, western blot studies were performed to assess the expression of insulin receptor beta (IR-β) upon insulin treatment (Fig. 2c). Interestingly, the IR-β expression decreased after 300 minutes of insulin incubation, and this is consistent with the gene expression data as shown in Fig. 2d.

### Time-series data analysis identifies 12 significant gene profiles/clusters in hCMEC/D3 monolayers after insulin treatment

**Fig. 3:**
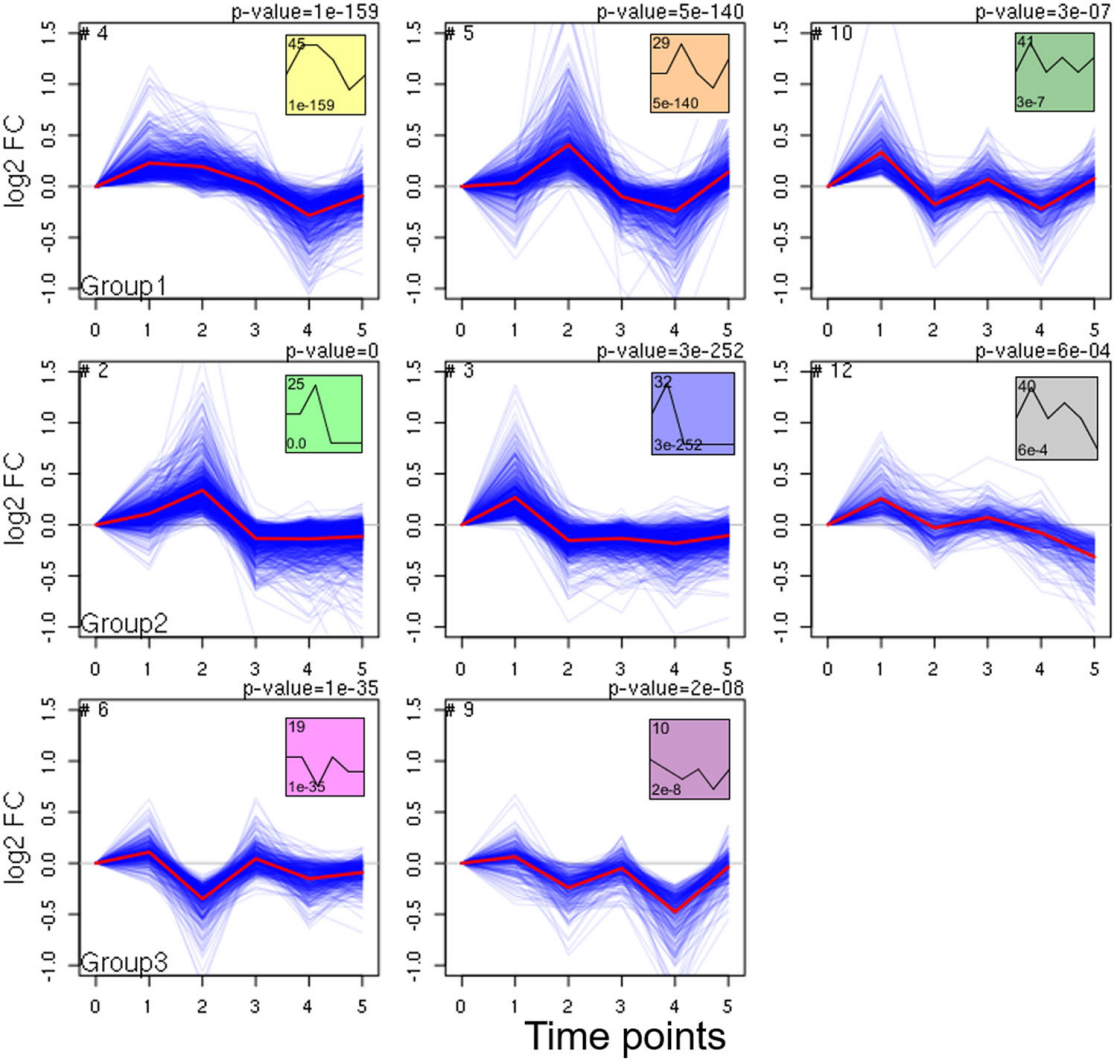
The significant clusters are divided into three groups based on the temporal change patterns and enriched pathways in each cluster. Inside each set (in the box), the cluster number is displayed in the left-hand corner and the corresponding profile defined by the STEM software is displayed in the right-hand corner. *p* value is presented on the top of each cluster. *p* value is determined by STEM software based on a binomial distribution by comparing the actual number of genes assigned to the group against the expected number of genes. After insulin treatment, gene expression went up in group 1, went up initially but decreased in group 2, and went down in group 3.

After removing the low expressed genes, we provided the normalized time-series gene expression data as the input to the STEM software^13^. The STEM method assigned all the genes to a set of pre-defined temporal expression profiles defined by the number of time points and maximum units of fold change. The STEM clustering method identifies distinct and representative temporal expressions of a collection of genes. All of the model profiles begin at zero, and the model profile is expected to increase, decrease, or remain stable (do not change) between two-time points. As shown in Supplementary Fig. 2, STEM software identified 12 significant clusters (colored) out of 50 profiles, with 6880 genes across the five-time points that display various patterns. The number of genes and *p* values of each significant cluster are presented in Table 1. The correspondence between cluster number as used herein and profile number in STEM software is displayed in Supplementary Table 1. For each cluster, the *p* value is determined based on a permutation test by comparing the number of genes assigned to a particular profile and genes that are expected to follow that specific profile by chance. After a thorough investigation of the individual clusters, we separated the clusters into 3 groups: clusters 4, 5, 10 consisting of a total of 1685 genes (from here on, we will refer to them as Group 1), clusters 2, 3, 12 consisting of a total of 2279 genes (Group 2) and clusters 6, 9 with 758 genes (Group 3) (Fig. 3). The grouping was based on two criteria. First criterion: clusters that followed similar profiles or dynamic trends were grouped together for further analysis. For example, after insulin treatment, the gene expression increased in group 1; went up rapidly but decreased at later time points in group 2; and decreased overall in group 3. Second criterion: functional annotation of genes in each cluster was conducted and the gene ontology pathways enriched were found to be similar among the clusters within one group (Supplementary Table 2). We retrieved the genes from each group and investigated the function of genes and pathways represented by these three groups or 8 clusters that demonstrated contrasting trends. Four other clusters were not grouped because no specific trend was observed and the enriched pathways in these clusters were not directly relevant to BBB or AD pathophysiology.

**Table 1:** Time-series clustering of the genes based on gene expression profile changes was conducted using the STEM software.

Time-series clustering	Number of genes	*p* value
Cluster 1	1185	0.0
Cluster 2	1115	0.0
Cluster 3	902	3e-252
Cluster 4	674	1e-159
Cluster 5	667	5e-140
Cluster 6	458	1e-35
Cluster 7	391	5e-19
Cluster 8	376	2e-14
Cluster 9	344	2e-8
Cluster 10	300	3e-7
Cluster 11	206	4e-4
Cluster 12	262	6e-4

STEM detected 12 significant gene clusters based on similar gene expression profiles across five-time points. The number of genes and *p* values in each cluster are listed.

### Functional annotation of genes in each group affected by insulin treatment shows the genesets involved in AD pathophysiology

We obtained the genes from each group (group 1 = 1685, group 2 = 2279, and group 3 = 758 genes) and conducted functional annotation using the Webgestalt software. Gene ontology (GO) enrichment analysis was performed on the genes in group 1 that were upregulated after insulin treatment. The genes are highly enriched in various biological processes such as the regulation of membrane lipid distribution, extracellular structure organization, and angiogenesis. Similarly, the GO enrichment analysis was conducted on the genes in group 2 that were upregulated initially after insulin treatment but decreased later. These genes are associated with biological processes related to the actin cytoskeleton, such as actin cytoskeleton reorganization, regulation of actin filament-based process, maintenance of location, and regulation of supramolecular fiber organization. In the last group where the gene expression decreased upon insulin exposure, enriched GO pathways are mostly inflammation-related, including response to interferon-beta, interferon-gamma, response to type I inflammation, and response to the virus. The GO pathways enriched in each group, representative genes, and their association with AD-related pathologies are listed in Table 2.

**Table 2:** GO enrichment analysis of genes in 3 groups and representative genes involved in AD pathology.

Group	Clusters	GO pathways	Gene symbols	Involvement in AD	Ref.
1	4, 5, 10	Regulation of membrane lipid distribution; Epithelial cell development; Extracellular structure organization; Tissue migration; Angiogenesis; Regulation of vascular development	LAMB2	Laminin inhibited Aβ fibrillation	^20,21^
			COL4A5 COL4A1 COL4A2	Collagen IV inhibited Aβ40 fibril formation.	^19^
			LEPR	Neuronal leptin receptor expression in the old 5XFAD brain was diminished	^69^
			VEGFB	VEGF improved memory and reduced Aβ levels.	^22^
			BMP1	BMP7 prevented neuronal injuries in PC12 cells induced by Aβ	^70^
2	2, 3, 12	Actin cytoskeleton reorganization; Regulation of actin filament-based process; Maintenance of location; Actin filament organization; Regulation of cytoskeleton organization	FLNA	Altered filamin A enables tau phosphorylation	^71^
			CDK5	Cdk5 regulates endothelial cell migration and angiogenesis	^72^
			MYH9	Myosin IIb regulates actin dynamics during synaptic plasticity and memory formation	^73^
			MYO1C		
			MYO5A		
3	6, 9	Response to interferon-beta; Response to type I interferon; Response to interferon-gamma; Response to virus; I-kappaB kinase/NF-kappaB signaling	IFITM2	IFITM3 is increased in AD mouse models; increases production of Aβ	^36^
			IFITM3		
			CASP1	Inhibition of caspase restored memory impairment in AD mouse modes	^38^
			ICAM-1	ICAM-1 expression is increased in AD brain	^39^
			LGALS9	Overexpression of Gal-3 enhances Aβ oligomerization	^74^

After insulin exposure, gene expression increased in group 1, increased initially but decreased at a later time in group 2, and decreased in group 3.

### Canonical pathway analysis has identified pathways that are critical for vascular functions

The genes in each group were further used to identify the known canonical pathways that are associated with the grouped genes using ingenuity pathway analysis (IPA) methods. The genesets (group 1 = 1685, group 2 = 2279, and group 3 = 758) were submitted to the IPA software, and the pathways with a *p* value <0.05 were identified. Because the IPA software is knowledge-based and consists of findings more from cancer research, the pathways were further screened based on their connection to vascular/AD pathologies in the literature reports. The top canonical pathways are shown in Table 3 with respective *z* scores; if the *z* score >1, the pathway is deemed to be activated after insulin treatment, and if *Z* score <−1, the pathway is considered inhibited after insulin treatment. The ratio column in Table 3 indicates the ratio of genes from the gene set (in this case, the groups) that map to a canonical pathway to the total number of genes present in that pathway.

**Table 3:** Ingenuity pathway analysis of the genes from each group.

Group	Ingenuity canonical pathways	*z* score	*p* value	Ratio
1	Glioblastoma multiforme signaling	3.051	0.000110	0.123
	GP6 signaling pathway	3.873	0.000513	0.126
	Factors promoting cardiogenesis in vertebrates	3.357	0.001288	0.113
	Inhibition of matrix metalloproteases	1.342	0.002692	0.179
	VEGF family ligand-receptor interactions	3.162	0.002754	0.131
	HGF signaling	3.317	0.005623	0.106
	Glioma signaling	2.646	0.008128	0.105
	Endothelin-1 signaling	3.5	0.013804	0.089
2	Autophagy	3.507	0.0000000	0.202
	Insulin receptor signaling	2.785	0.0000000	0.221
	CDK5 signaling	1.964	0.0000016	0.214
	NGF signaling	4.69	0.0000138	0.195
	IGF-1 signaling	3.13	0.0000186	0.202
	Senescence pathway	4.217	0.0000324	0.141
	PTEN signaling	−3.545	0.0000347	0.173
	AMPK signaling	4.536	0.0000398	0.149
3	Interferon signaling	−3.162	0.0000000	0.278
	Oxidative phosphorylation	−3	0.0006310	0.0818
	Integrin signaling	−2.828	0.0023988	0.0563
	Neuroinflammation signaling pathway	−3.464	0.0037154	0.0476
	Th1 pathway	−1.342	0.005011872	0.0656
	Leukocyte extravasation signaling	−2.646	0.0093325	0.0518
	PDGF signaling	−2.449	0.0107152	0.0698
	NRF2-mediated oxidative stress response	−2	0.0338844	0.0497

List of pathways, with *z* score, *p* values, and the ratio are listed above. Pathways are ordered by *p* values.

**Fig. 4:**
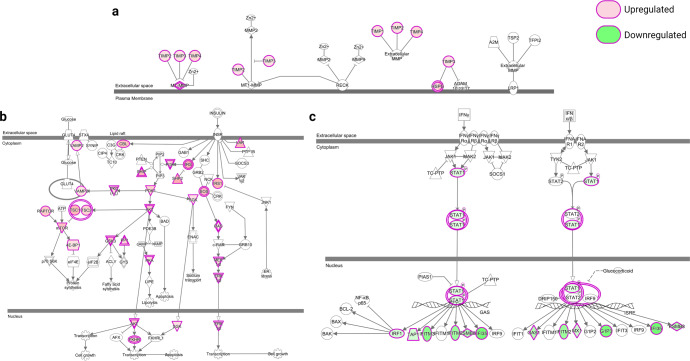
Gene expression changes in the representative pathway of each group. **a** Inhibition of matrix metalloproteases (enriched in Group 1). **b** Insulin receptor signaling (enriched in Group 2). **c** Interferon signaling (enriched in Group 3). Downregulated genes are indicated with green shading and upregulated genes in red.

Gene expression changes of representative pathways in each group were displayed in Fig. 4. Inhibition of matrix metalloproteases pathway (enriched in group 1) was activated by insulin treatment and expression of all tissue inhibitor of metalloproteinase (TIMPs) genes increased. The insulin receptor signaling pathway (enriched in group 2) was activated by insulin at early time intervals, and critical genes such as PI3K, AKT, RAS, ERK were upregulated. Interferon signaling (enriched in Group 3) was inhibited by insulin treatment with downregulation of STAT1, IFITM2, G1P3, and others.

## Discussion

The BBB is a critical gatekeeper that dynamically removes toxic metabolites from the brain and delivers essential nutrients, critical proteins, and growth factors to maintain brain functions. BBB dysfunction resulting in reduced cerebral blood flow, impaired amyloid-beta clearance, and cerebrovascular inflammation promotes AD progression. Notably, several of these pathological features are consistently manifested in type-2 diabetes mellitus (T2DM), which is recognized as a major risk factor for AD^14,15^. However, the pathophysiological connections between insulin resistance and BBB dysfunction in AD are only partially understood. Identifying insulin-responsive pathways in the BBB endothelium will allow us to elucidate their role in various BBB functions and assess the impact of insulin resistance on BBB dysfunction.

### GO enrichment analysis

In the current study, gene expression changes in polarized hCMEC/D3 monolayers following insulin treatment were clustered by STEM analysis. Twelve clusters were identified to be significant and further classified into three groups based on the dynamics of their regulation. In the first group, the gene expression increased after insulin treatment, whereas in the second group, the gene expression increased initially but decreased with a longer duration of insulin exposure. In the last group, the gene expression decreased after insulin treatment. The biological functions of the genes from each group were retrieved and further evaluated using GO enrichment analysis.

In the first group, where gene expression increased upon insulin exposure, pathways regulating membrane lipid distribution, extracellular structure organization, and angiogenesis are significantly enriched. These pathways were shown to be involved in vascular development^16–18^. The BBB breakdown has been identified as one of the earliest changes in the AD brain that occurs before dementia and brain atrophy^4,5^. The BBB disruption enables the brain entry of blood-derived cells, pathogens, and other products associated with inflammatory response and activation of neurodegenerative pathways. The current results demonstrated that insulin could activate pathways that are important for BBB formation and maturation. It can be envisioned that these pathways can be deactivated during insulin resistance, which is prevalent in AD, thereby inducing BBB disruption.

Further, critical genes associated with AD pathogenesis were found in the first group, and the expression of those genes increased after insulin exposure, one of which is collagen type IV proteins alpha genes (COL4A). Type IV collagen proteins are integral components of basement membranes that support the structure and function of NVU. Moreover, collagen IV was shown to inhibit the aggregation of Aβ40^19^, which is a major contributor to cerebrovascular amyloid angiopathy (CAA) in AD. Similarly, the expression of laminin subunit beta 2 (LAMB2) is increased by insulin, and laminin inhibits the fibrillization of both Aβ40^20^ and Aβ42^21^. Insulin also increases the expression of the vascular endothelial growth factor B (VEGFB) gene, which belongs to the first group. The VEGF family members regulate the formation of blood vessels and have been shown to improve memory and reduce Aβ levels in transgenic mouse models of AD^22^.

In the second group, the gene expression increased at early time points but decreased with longer insulin exposure. Enriched GO pathways include actin cytoskeleton reorganization, regulation of actin filament-based process, maintenance of location, and regulation of supramolecular fiber organization. The actin cytoskeleton is involved in various critical cellular processes such as cell migration and structural support. As a dynamic interface between brain and blood, BBB is responsible for the transport of cargo in both directions, which is regulated by actin-controlled processes in endothelial cells. A branched actin network promotes endocytic internalization by harnessing elastic energy stored in bent filaments^23^. During intracellular trafficking, myosin is recruited to the cargo containing vesicles to mobilize them on actin filament trackers^24^. Actin cytoskeleton can also facilitate fusion events of vesicles thus contributing to the trafficking of cargos and exocytosis^25^. In addition to the trafficking function, actin was also found to regulate BBB permeability because treatment of actin inhibitor cytochalasin B was shown to increase cerebrovascular permeability to horseradish peroxidase in rats^26^. Numerous studies of human postmortem tissues and animal models indicate the pathways regulating actin cytoskeleton stability are disrupted in AD^27^. Thus insulin could potentially mitigate these pathological effects and increase the expression of genes that are critical for actin-based processes in endothelial cells that regulate the barrier integrity and transport network of the BBB. However, the expression of these genes was observed to decrease at later time points when the cells were exposed to insulin for an extended period. One potential reason is that the long-term insulin exposure triggers insulin resistance which is associated with molecular changes including degradation of insulin receptor^28^, inhibitory phosphorylation of insulin receptor substrate^28^, and inhibition of pAKT and pERK^29^. Indeed, our western blot study found that the protein expression of IR-β in BBB endothelial cells was substantially reduced after 300 minutes of incubation with insulin **(**Fig. 2c**)**. Thus, insulin resistance induced by long-term insulin exposure may be responsible for dampening or even reversing insulin response. However, the molecular mechanisms by which insulin resistance affects gene expression and regulates pathways reported in the current study warrants further investigation.

In the last group, however, the gene expression decreased rapidly upon insulin exposure. The most enriched GO pathways of this group are connected to inflammation, such as response to interferon-beta, interferon-gamma, response to type I inflammation, and response to the virus. Inflammation is a common pathological feature of both AD and diabetes^30^, mediated by diverse immune cells and pro-inflammatory cytokines such as interleukin-1 (IL-1), tumor necrosis factor-alpha (TNF-α), and interferons. Insulin has been shown to reduce inflammation in peripheral tissues and the brain^31–33^. However, the anti-inflammatory effect of insulin on BBB endothelial cells and the underlying mechanisms are not well understood. Previous studies have demonstrated that type I interferon response drives neuroinflammation and synapse loss in AD patients^34^, and higher levels of interferon-gamma were observed in patients with mild cognitive impairment (MCI) compared to controls^35^. The current study suggested that one possible mechanism by which insulin reduces inflammation is to decrease the expression of genes in interferon-related pathways. Further, several genes that constitute group 3 are shown to contribute to AD progression, one of which is interferon-induced transmembrane protein 3 (IFITM3). A recent study demonstrated that the protein encoded by this gene is upregulated with aging and in mouse models of familial AD. It was also shown that IFITM3 could bind to γ-secretase in neurons and astrocytes and increase its activity to produce Aβ peptides^36^. Another example is caspase 1 (CASP1), which is involved in cell apoptosis and activation of IL-1. Inhibition of caspase 1 by chemical inhibitor VX-765 reduced Aβ deposition and improved spatial memory in AD mouse models^37^. Using the model of BBB injury, induced by organophosphate paraoxon, Israelov et al.^38^ demonstrated that caspase inhibition could restore the BBB integrity and reduce the inflammatory response. These studies indicate that caspase 1 could be a target upon which insulin acts to reduce inflammation and prevent BBB breakdown during AD. Intercellular adhesion molecule 1 (ICAM-1), an inflammatory endothelial marker, was found to be upregulated in the cerebrovascular endothelium of AD patients^39^. Insulin was shown to decrease ICAM-1 expression in human aortic endothelial cells^40^, which is consistent with the current observation that the ICAM-1 gene (group 3) is downregulated in hCMEC/D3 monolayers upon insulin exposure.

### IPA canonical pathway analysis

The genes from each group were further investigated using Ingenuity Pathway Analysis (IPA) software to identify known signaling pathways. The expression of genes in the first group was activated by insulin exposure; the GO analysis demonstrated that these genes prevented BBB breakdown in AD. Similarly, the IPA analysis has shown that the insulin treatment upregulated signaling pathways that promote vascular development such as glioma invasiveness signaling, glioblastoma multiforme signaling, factors promoting cardiogenesis in vertebrates, and inhibition of matrix metalloproteases (Table 3). One of the major gene families present in these pathways is the tissue inhibitor of metalloproteinases (TIMPs). All members of TIMPs are upregulated by insulin treatment, including TIMP1, TIMP2, TIMP3, and TIMP4. The TIMPs are the natural tissue inhibitors of matrix metalloproteinases (MMPs), which degrade various extracellular matrix components. Therefore, inhibition of MMPs by TIMPs might protect against vascular degeneration, especially in brain diseases. Indeed, a previous study has demonstrated that TIMP1 could inhibit MMP-9 expression and prevent BBB disruption and neuronal apoptosis after ischemic injury^41^. In postmortem AD brains, MMP-9 was found to express in close proximity to extracellular amyloid plaques^42^ and mediate tau aggregation^43^, inflammation^44^, and BBB disruption^45^. However, the therapeutic effect of TIMPs on AD pathologies, especially on vascular dysfunction, has been underexplored. Our results indicated that insulin could upregulate TIMPs expression and thereby prevent BBB breakdown and other vascular changes associated with AD. In addition to MMPs, type IV collagenase was also shown to be inhibited by TIMP2 and the inhibition could reduce the proteolytic opening of BBB^46^. The GP6 signaling pathway was also found in the first group and upregulated after insulin treatment. This pathway contains many collagens and laminins that form the basement membrane of the BBB. For instance, collagen VI in this pathway was previously reported to prevent the neurotoxicity engendered by Aβ^47^. Insulin exposure was previously shown to increase the expression of tight junctional proteins in BBB endothelial cells^48^. Additionally, our findings suggest that insulin could also enhance the integrity of the basement membrane. By promoting these changes, insulin could restore BBB integrity and mitigate BBB dysfunction.

The second group, where gene expression increased rapidly upon shorter insulin exposure but decreased upon more prolonged exposure, includes several significantly enriched pathways such as insulin receptor signaling, NGF signaling, and IGF-1 signaling. These pathways were found to be upregulated with a *z* score >1 when the gene expression data at early time points were subjected to IPA analysis (Table 3). The results are physiologically reasonable because insulin is well known to activate these growth factor-stimulated pathways and promote cell proliferation and survival. However, when the gene expression data were subjected to IPA analysis at later time points, the insulin receptor signaling pathway was downregulated (Supplementary Table 3). One plausible explanation for this biphasic phenomenon is that long-term exposure to insulin could induce insulin resistance, thus deactivating the insulin signaling pathway. Cyclin-dependent kinase-5 (CDK5) signaling pathway was also enriched in this group and upregulated by insulin, which agrees with a previous study showing that insulin activated Cdk5 and mediated glucose uptake in adipocytes^49^. Further, Cdk5 is shown to colocalize with F-actin in neurons^50^ and control actin dynamics by activating actin-binding proteins such as cofilin^51^. These findings suggest that Cdk5 could be a potential mediator through which insulin activates actin-based processes and regulates the trafficking and barrier function of BBB, as discussed above in the GO enrichment analysis. On the other hand, another enriched pathway, phosphatase and tensin homolog (PTEN) signaling, was downregulated by insulin treatment with a *z* score <−1. It was previously shown that PTEN protein levels are increased upon insulin receptor inhibition, whereas the PTEN expression is reduced upon insulin signaling activation^52^. Further, inhibition of PTEN was shown to improve synaptic function and cognition in both in vitro and in vivo models of AD^53^. Given that intranasal insulin administration was shown to improve cognition in AD patients^54^, our finding provided a potential mechanism by which insulin exerts such beneficial effects.

In the last group where insulin treatment decreased gene expression, the IPA analysis identified several biological pathways that are downregulated. These pathways include inflammation-related pathways, such as interferon signaling, antigen presentation pathway, oxidative phosphorylation, and neuroinflammation signaling pathway. The C-C Motif Chemokine Ligand 2 (CCL2) is a common gene in these pathways whose expression was inhibited by insulin. Multiple lines of evidence have shown that CCL2 could promote AD progression. For example, a higher level of CCL2 in CSF was shown to be associated with faster cognitive decline during the early stages of AD^55^. In another study, CCL2 overexpression in the brain activated glial cells and promoted tau pathology in mouse models^56^. The mitochondria dysfunction pathway was also enriched in this group and downregulated by insulin exposure. Mitochondrial damage has been proposed as a major contributor to AD pathogenesis as healthy mitochondria support critical neuronal activities and prevent brain cells from oxidative stress^57,58^. Further, cerebrovascular endothelial mitochondria dysfunction has been reported in CAA and aggravates disease progression^59^. However, the crosstalk between insulin signaling and mitochondrial dysfunction pathways is yet to be elucidated. Our results demonstrated that insulin triggers pathways that could potentially prevent mitochondria dysfunction in BBB endothelial cells and provide novel directions for future research.

In conclusion, we conducted a time-series RNA-Seq analysis of insulin-treated hCMEC/D3 cell monolayers to decipher insulin-responsive pathways in the BBB endothelium. The pathway analyses identified biological processes and signaling pathways that are activated or inhibited by insulin treatment, and these pathways were found to modulate BBB integrity and function. These findings unravel the dynamics of insulin action on the BBB endothelium and allows for the identification of critical molecular mediators and pathways associated with BBB dysfunction. We will further investigate how these pathways are disrupted in AD, especially in patients with T2DM. While this study has mainly focused on capturing the transcriptomic changes upon insulin treatment, further experiments are needed to interrogate if they are translated to changes in protein expression. From the resultant data, insulin-responsive genes/proteins that regulate BBB integrity and function could be identified for functional validation studies.

## Methods

### Cell culture and Illumina TruSeq v2 mRNA protocol

**Fig. 5:**
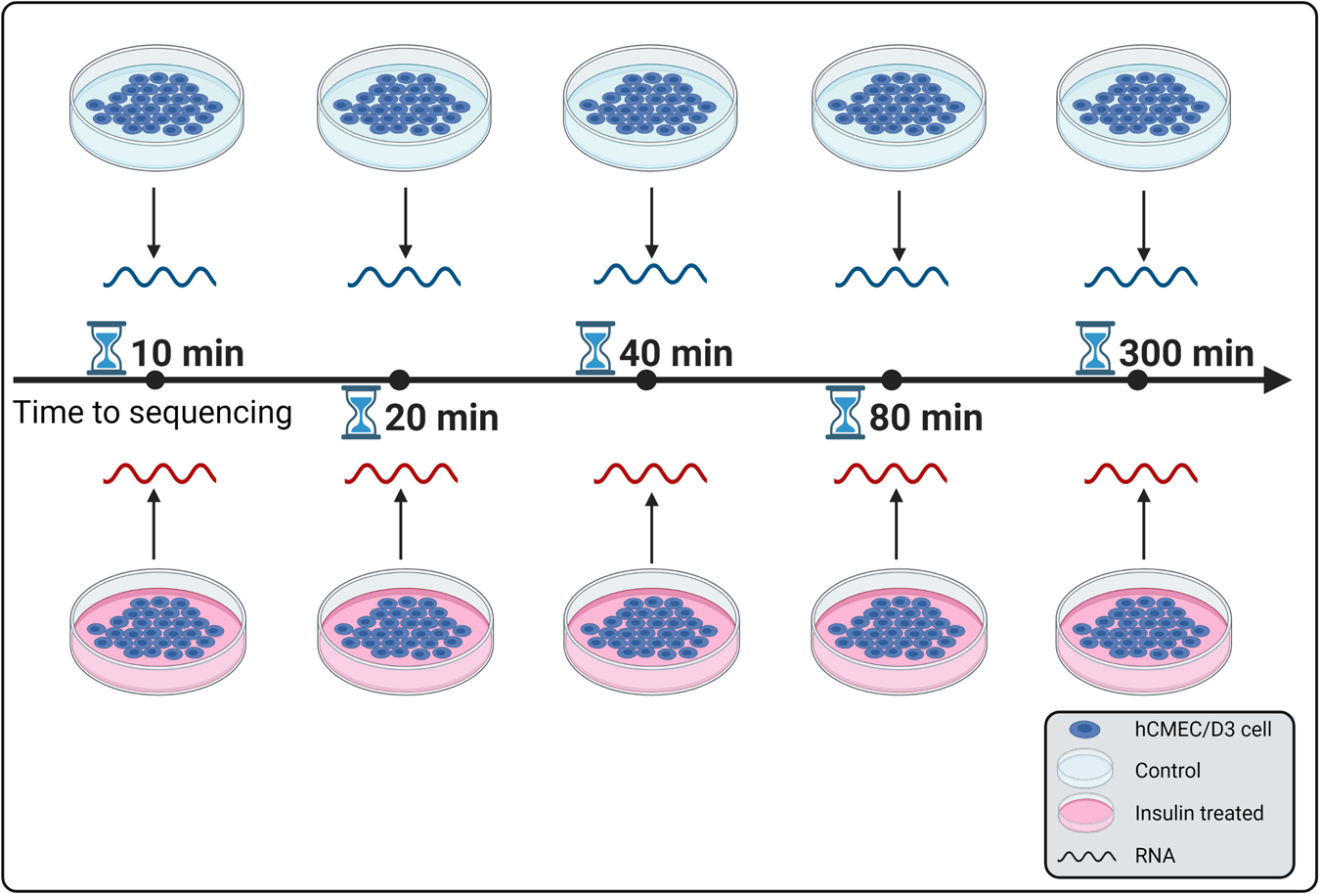
RNA-Sequencing experimental design. Human cerebral microvascular endothelial cell (hCMEC/D3) monolayers were treated with and without insulin for 10, 20, 40, 80 and 300 minutes. Paired-end RNA libraries from control and insulin-treated cell monolayers were then prepared.

The hCMEC/D3 cell line was kindly provided by P-O Couraud (Institut Cochin, France). The cells were seeded at 50,000 cells/cm^2^ on 24 mm Transwell^®^ inserts (Corning Inc., Corning, NY) precoated with collagen and cultured under 5% CO_2_ at 37 °C in endothelial cell growth basal medium-2 (Lonza, NJ) containing 5% of fetal bovine serum. Various supplements including 1 ng/ml human basic fibroblast growth factor (PeproTech, NJ), 1% chemically defined lipid concentrate (Gibco, NY), 10 mM HEPES, 5 µg/ml ascorbic acid, 1.4 µM hydrocortisone, and 1% penicillin-streptomycin (MP Biomaterials, OH) were added to the cell culture medium. A confluent and polarized monolayer was formed after culturing for seven to nine days, as observed under the microscope. Based on our previous studies, polarized endothelial cell monolayers formed under these conditions exhibited transendothelial electrical resistance values ranging between 80 and 120 Ω/cm^2^. The experiments were conducted as outlined in our previous publication^60^, where we presented a comprehensive transcriptomic analysis (>900 million reads) of untreated hCMEC/D3 monolayers. In this study, the same BBB model was treated with 100 nM insulin at various time points (*t* = 10, 20, 40, 80, and 300 minutes). This insulin concentration has been widely used for in vitro investigations and was shown to activate downstream signaling pathways in endothelial cells^61–63^. We also harvested synchronized control hCMEC/D3 cell monolayers without insulin treatment at the respective time points, as shown in Fig. 5. Paired-end RNA libraries from insulin-treated and control hCMEC/D3 cell monolayers were prepared according to the manufacturer’s instructions using TruSeq RNA Sample Prep Kit v2 (Illumina, San Diego, CA). Briefly, poly-A mRNA was purified from total RNA using oligo dT magnetic beads for ribosomal RNA depletion. Double strand cDNA was synthesized and enriched by 12 cycles of PCR. The resultant average RNA integrity number was 8.3 (±0.7). The libraries were sequenced as 51 × 2 paired-end reads on an Illumina HiSeq 2000 using TruSeq SBS sequencing kit (version 3) and HCS data collection software (version 2.0.12.0)^60^. Base-calling was performed using Illumina’s RTA (version 1.17.21.3).

### Cell viability assay

The cell viability was assessed using the Live/Dead staining viability/cytotoxicity Kit (Molecular Probes, Inc., Eugene, OR) according to the manufacturer’s instructions. In brief, a combination of calcein-AM and ethidium homodimer-1 (EthD-1) was used to stain the cells. Calcein-AM is well retained by live cells and is metabolically converted by intracellular esterase to produce a green fluorescence. EthD-1 is excluded from live cells but enters dead cells and produces a red fluorescence upon binding to nucleic acids. After incubation with insulin, the cells were trypsinized, resuspended in PBS, and were incubated with the dyes at room temperature for 15–20 minutes. The stained cells were analyzed by flow cytometry.

### Western blotting

Following the treatment with insulin for 40 and 300 minutes, the cells were washed three times with PBS and lysed with RIPA buffer containing protease and phosphatase inhibitors (Sigma-Aldrich, St. Louis. MO). Total protein concentrations in the lysates were determined by bicinchoninic acid (BCA) assay (Pierce, Waltham, MA). The lysates (25 µg protein per lane) were loaded onto 4-12% Criterion XT precast gels and proteins were separated by SDS-PAGE under reducing conditions (Bio-Rad Laboratories, Hercules, CA). The proteins were then electroblotted onto a 0.45 µm nitrocellulose membrane, blocked with 5% nonfat dry milk protein (Bio-Rad Laboratories, Hercules, CA), and incubated overnight at 4 °C with primary antibodies against IR-β (1:1000, #3025, Cell Signaling Technology, Denvers, MA) and GAPDH (1:1000, #5174, Cell Signaling Technology, Danvers, MA). Afterwards, the membrane was incubated with IR-dye conjugated secondary antibody (1:2000, catalog #LIC-926-32211) for 1 h at room temperature. All blots were derived from the same experiment and were processed in parallel. Immuno-reactive bands were then imaged (Odyssey CLx; LI-COR Inc, Lincoln, NE) and the band intensities were quantified by densitometry (Image StudioTM Lite Software, LI-COR Inc, Lincoln, NE). Uncropped scans of the blots were presented in Supplementary Fig. 3.

### RNA-Seq data processing and gene expression quantification

After paired-end transcriptome sequencing, the RNA-Seq data of the control and treated samples were processed using the MAP-RSeq to obtain various genomic features from the RNA-Seq experiment. The MAP-RSeq pipeline, a comprehensive computational workflow developed at the Mayo Clinic^64^, allowed us to obtain multiple genomic features, such as gene expression, exon counts, and fusion transcripts from RNA-Seq data. MAP-RSeq also provided quality control reports and summary statistics of the sequencing reads. The reads were mapped to the human genome reference hg19 build. Then the total number of reads mapped reads, reads mapped to the genome, and the numbers of reads mapped to junctions were obtained for each sample. Gene expression counts were quantified using the HTSeq module (http://www-huber.embl.de/users/anders/HTSeq/doc/count.html) from MAP-RSeq pipeline for control hCMEC/D3 monolayers and matched insulin-treated hCMEC/D3 monolayers. Principal component analysis and gene expression data analysis for five treatment and control pairs were conducted using the R programming language. The bioinformatics and statistics workflow was presented in Supplementary Fig. 4.

### Time-series gene expression analysis

Normalized gene expression data was used for time-series data analysis. We determined the difference between the paired samples (insulin-treated versus control hCMEC/D3 monolayers) at all five-time points for every gene. The resulting gene expression differences were then provided as input to the Short Time-series Expression Miner (STEM)^13^ method to identify clusters of genes with distinct and representative patterns. STEM is an application specifically designed for the clustering analysis of short time-series gene expression data (3-8 time points). The STEM method identifies profiles/clusters of statistically significant genes based on the correlation coefficient (the minimal default correlation is 0.7). In the STEM software, we chose parameter options: the maximum number of model profiles = 50, a maximum unit change in model profiles between time points = 2, and the clustering method = STEM. The significance of a cluster is determined by STEM software based on a binomial distribution by comparing the actual number of genes assigned to the group against the expected number of genes.

### Gene set and pathway analysis

The top profiles/clusters of genes identified by the STEM software were retrieved and used to conduct overrepresentation analysis using the webGestalt software and gene ontology database^65^. Pathway analysis of genes from the significant clusters was performed using Ingenuity pathway analysis software (QIAGEN Inc., https://www.qiagenbioinformatics.com/products/ingenuitypathway-analysis) to predict pathways that are activated or inhibited after insulin treatment.

## Supplementary information

Supplementary information

## Data Availability

The data are available in the GEO database and the accession ID is GSE195781.

## Code availability

The code is available in the github repository: https://github.com/KalariRKLab/InsulinTreatedBBB.
