# Supplementary material for: Mapping the dynamics of insulin-responsive pathways in the blood–brain barrier endothelium using time-series transcriptomics data
Source: NPJ Syst Biol Appl. 2022 Aug 16;8:29. doi: 10.1038/s41540-022-00235-8 (PMC9381797; doi:10.1038/s41540-022-00235-8)
Supplement: Supplementary file 1 — Supplementary information [file 41540_2022_235_MOESM1_ESM.pdf]

Supplementary information

Mapping the dynamics of insulin-responsive pathways in the blood-brain barrier endothelium using time-series transcriptomics data

Zengtao Wang[1, *], Xiaojia Tang[2, *], Suresh K. Swaminathan[1], Karunya K. Kandimalla[1, $], Krishna R. Kalari[2, $]

[1]Department of Pharmaceutics and Brain Barriers Research Center, College of Pharmacy, University of Minnesota, MN, USA

[2]Department of Quantitative Health Sciences, Mayo Clinic, 200 First Street SW, Rochester, MN USA

*** Authors contributed equally**

[$]- Co-Corresponding Authors

Krishna R. Kalari

Department of Quantitative Health Sciences,

Mayo Clinic, Rochester

MN 55901, USA.

Tel: 507-538-4602 Email: Kalari.krishna@mayo.edu

Karunya K. Kandimalla,

Department of Pharmaceutics and Brain Barriers Research Center,

College of Pharmacy,

University of Minnesota, Minneapolis,

MN 55455, USA.

Tel: 612-624-3715; Email: kkandima@umn.edu

Supplementary Figures

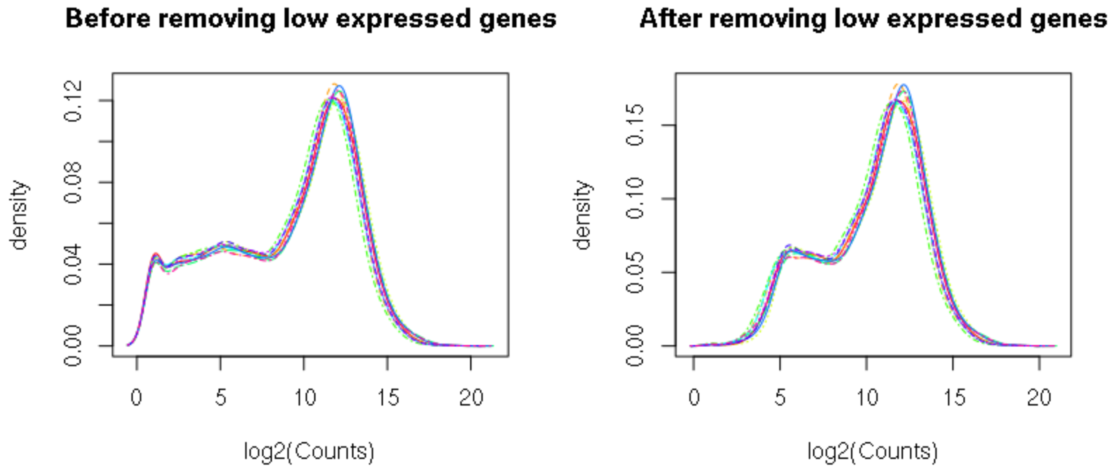

Supplementary Figure 1. The density plot of the raw counts and filtered counts of all 10 samples (after removing low expressed genes with <32 counts of all samples). It can be seen that the raw counts (left) has a peak around 0 (not expressed gene) and a wide peak around 32 (relatively low expressed genes, $\log_2 32 = 5$). After removing the genes that were not expressed (raw counts <32 in all samples, right panel), the peak around 0 was gone and a more defined peak for those relatively low expressed genes.

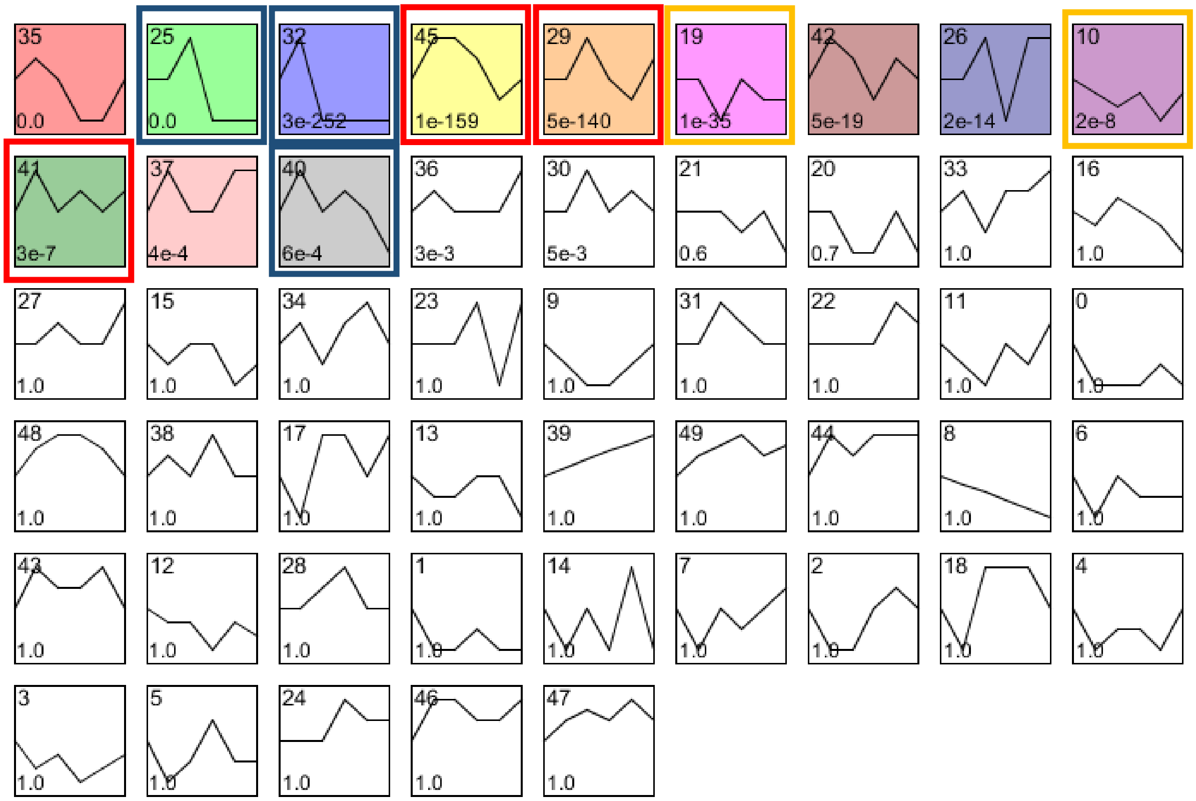

Supplementary Figure 2. 50 gene profiles defined by STEM that display various temporal patterns. 12 profiles were found to be significant (colored). Profiles are ordered based on the p-value (shown in the bottom-left corner). The profile number is displayed in the top-left corner. 8 significant profiles are divided into three groups based on the temporal change patterns and enriched pathways. Group1 (red box): profile 45, 29, 41; Group2 (blue box): profile 25, 32, 40; Group3 (yellow box): profile 19, 10. In each profile, the x axis indicates the time points, y axis is the log fold change of gene expression.

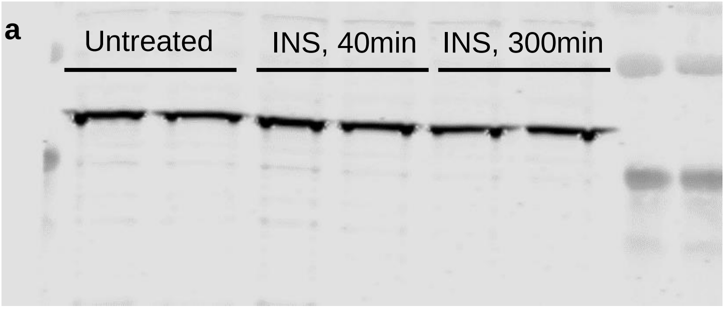

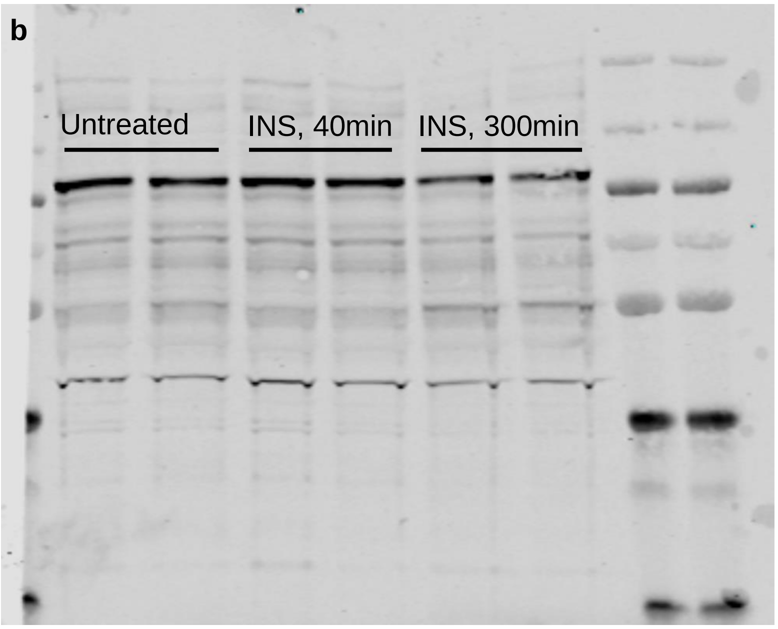

Supplementary Figure 3. Uncropped scans of western blots. (a) GAPDH (b) Insulin receptor-β

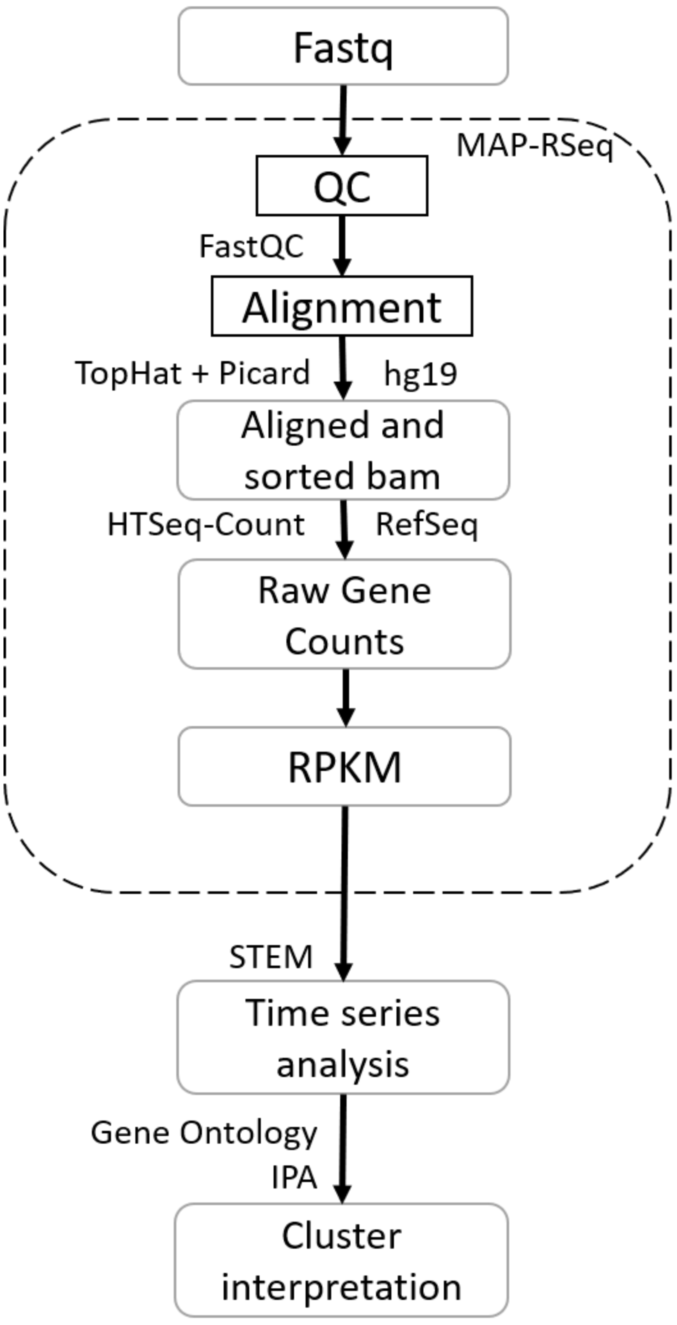

Supplementary Figure 4. Flow chart demonstrating steps performed in the bioinformatics analysis. All software used were notated in the flowchart.

Supplementary tables

Supplementary Table 1. Correspondence between cluster number as used in main manuscript and profile number in STEM software

Profile number in STEM	Cluster number in main manuscript
35	1
25	2
32	3
45	4
29	5
19	6
42	7
26	8
10	9
41	10
37	11
40	12

Supplementary Table 2. GO enrichment analysis of genes in each cluster.

Group	Cluster	Top GO pathways
1	4	extracellular matrix structural constituent
		basement membrane
		blood vessel development
		cardiovascular system development
		vasculature development
	5	basolateral plasma membrane
		anatomical structure formation involved in morphogenesis
		blood vessel development
		cardiovascular system development
		vasculature development
	10	epithelial cell development
		establishment of protein localization to plasma membrane
		sprouting angiogenesis
		angiogenesis
		blood vessel endothelial cell migration
2	2	cytoskeleton organization
		cytoskeleton
		movement of cell or subcellular component
		microtubule-based process
		microtubule cytoskeleton
	3	regulation of actin filament-based process
		regulation of actin cytoskeleton organization

		actin nucleation
		regulation of actin polymerization or depolymerization
		actin filament polymerization
	12	actin cytoskeleton
		exocytic vesicle membrane
		transport vesicle membrane
		cell projection organization
		regulation of regulated secretory pathway
3	6	cellular response to type I interferon
		type I interferon signaling pathway
		response to type I interferon
		immune response
		cytokine-mediated signaling pathway
	9	response to interferon-gamma
		cellular response to interferon-gamma
		defense response
		interferon-gamma-mediated signaling pathway
		innate immune response

Supplementary Table 3. Ingenuity pathway analysis of the gene expression from group 2 at later time points

Ingenuity Canonical Pathways	z-score	p-value	Ratio
Autophagy	-3.202	0.0000000	0.202
Insulin Receptor Signaling	-2.414	0.0000000	0.221
CDK5 Signaling	-1.964	0.0000016	0.214
NGF Signaling	-4.69	0.0000138	0.195
IGF-1 Signaling	-2.683	0.0000186	0.202
Senescence Pathway	-3.904	0.0000324	0.141
PTEN Signaling	3.962	0.0000347	0.173
AMPK Signaling	-3.578	0.0000398	0.149
